# A putative pharmacodynamic ceiling for topical corticosteroids in atopic dermatitis: a steroid-primed inflammasome and caspase-1–mediated glucocorticoid receptor cleavage

**DOI:** 10.3389/fimmu.2026.1988621

**Published:** 2026-10-22

**Authors:** Seung-Yong Seong

**Affiliations:** 1 Department of Microbiology and Immunology, Seoul National University College of Medicine, Seoul, Republic of Korea; 2 Department of Biomedical Sciences, Seoul National University College of Medicine, Seoul, Republic of Korea; 3 Wide River Institute of Immunology, Seoul National University College of Medicine, Hongchon, Gangwon, Republic of Korea; 4 Shaperon Inc., Seoul, Republic of Korea

**Keywords:** atopic dermatitis, caspase-1, glucocorticoid receptor, NLRP3 inflammasome, P2X7 receptor, steroid-induced rosacea, topical corticosteroids, topical steroid withdrawal

## Abstract

**Background:**

Topical corticosteroids (TCS) remain the first-line anti-inflammatory treatment for mild-to-moderate atopic dermatitis (AD), yet continuous use is constrained by cutaneous toxicity, rebound phenomena and topical steroid withdrawal (TSW).

**Objective:**

This article proposes a mechanistic model in which glucocorticoids prime a cutaneous inflammasome axis that both sustains relapse risk and progressively reduces glucocorticoid receptor (GR) availability through caspase-1-mediated cleavage.

**Methods:**

A narrative, hypothesis-generating synthesis integrated clinical trials of proactive maintenance and non-steroidal topicals with mechanistic data on glucocorticoid signaling, NLRP3 inflammasome biology, purinergic P2X7 receptor (P2X7R) pathways, keratinocyte-immune crosstalk and GR regulation.

**Hypothesis:**

TCS are among the most effective topical agents for AD, but prolonged use is associated with steroid-associated rosacea-like dermatitis and TSW, iatrogenic phenotypes with no non-steroidal counterpart. Residual relapse under proactive maintenance is of comparable magnitude whether maintenance is steroidal or non-steroidal and is therefore taken here as the clinical problem to be solved. Glucocorticoids can transactivate NLRP3 in myeloid cells and induce CCL20 and TLR2 in keratinocytes, increasing inflammasome sensor abundance and sensitivity. On tapering or withdrawal, under continued subclinical type 2 inflammation, barrier disruption, microbial colonization and LL-37 and ATP release, the primed P2X7R-NLRP3 axis may discharge. In leukemia cells, active caspase-1 cleaves GR, reducing receptor occupancy at glucocorticoid-responsive elements and conferring steroid resistance; analogous activity in skin would be expected to narrow progressively the GR pool available to subsequent TCS doses.

**Conclusions:**

The model reframes steroid-associated rosacea-like dermatitis and TSW as predictable discharges of a pharmacologically primed inflammasome rather than as idiosyncratic toxicity, with the limit on prolonged TCS use proposed to be set by P2X7R-NLRP3-caspase-1-GR interactions. It favors an add-on strategy co-administered with TCS from induction onward, targeting inflammasome assembly or caspase-1 activity to preserve GR integrity and prolong safe first-line steroid use, rather than relying on second-line non-steroidal substitution alone. No new experiments, patient-level analyses or meta-analyses are presented; prospective studies should quantify inflammasome activation, extracellular ATP and GR cleavage in human skin across the induction, maintenance, tapering and withdrawal phases of TCS therapy.

## 1 Introduction

Topical corticosteroids (TCS) are widely accepted as first-line anti-inflammatory treatment for mild-to-moderate atopic dermatitis (AD) (1–3). In routine practice, mild-to-moderate AD is managed with regular emollient use and topical corticosteroid treatment of active lesions until adequate disease control is achieved, generally during a short induction course with reassessment within several weeks (2, 4–6). Once disease control has been achieved, guideline-supported proactive therapy consists of intermittent treatment of previously affected, flare-prone sites with a topical anti-inflammatory agent, commonly a corticosteroid applied twice weekly—while emollient therapy is continued (2, 4, 5, 7). It is within this induction-to-maintenance sequence that a duration limit restricting prolonged TCS exposure may become clinically relevant.

Multiple guidelines endorse TCS in this position. The American Academy of Dermatology strongly recommends TCS for adults with AD and supports intermittent medium-potency TCS as twice-weekly maintenance therapy (2). Chinese guidelines similarly designate TCS as first-line therapy (3), and European guidance recommends the same proactive approach (5). Surveys of international dermatologists confirm that TCS are viewed as the mainstay topical anti-inflammatory treatment and are commonly used proactively on previously involved sites (4).

Network meta-analyses reinforce this positioning. A Cochrane review of almost 300 randomized trials in eczema placed potent TCS, topical Janus kinase inhibitors (JAKi) and tacrolimus 0.1% among the most effective topical treatments (8). In these analyses, short-term TCS use at any potency did not significantly increase clinically apparent skin thinning, and atrophy remained uncommon even over extended treatment durations (8). More recent evidence syntheses underpinning joint American guidelines similarly found that mid-potency corticosteroids improved multiple patient-important outcomes with moderate-to-high certainty and ranked among the best topical therapies on composite benefit metrics, ahead of several non-steroidal agents (1).

Thus, TCS are not weak medications displaced by superior alternatives; rather, they are potent and effective agents constrained by a relatively narrow therapeutic window (1, 8, 9). This asymmetry-robust anti-inflammatory efficacy paired with concerns about duration-dependent safety and rebound-serves as the premise for this hypothesis article. Accordingly, this Hypothesis and Theory article explores potential receptor-level mechanisms underlying this limited responsiveness.

For this hypothesis article, a “pharmacodynamic ceiling” is an exposure-conditioned reduction in the response of viable skin cells to an equivalent local glucocorticoid exposure, relative to their pretreatment state or an appropriately matched control. “Ceiling” is used as conceptual shorthand for diminished responsiveness, not as a measured maximal dose–response plateau.

In practice, the major constraints on TCS use are tolerability, safety concerns, and fear of rebound. Guidelines restrict continuous TCS use to short-term courses and recommend dose adjustment or treatment modification if an adequate response is not achieved within around four weeks (6). The most prominent adverse effect is skin atrophy, which is potentiated by higher potency, occlusion, thin skin, and older age (9). Long-term application of potent TCS to facial skin can lead to confluent erythema, telangiectasia, and papulopustular eruptions that resemble rosacea and are frequently followed by characteristic rebound upon discontinuation (10, 11); steroid-associated rosacea has been reported to differ from non-steroid-associated rosacea in eyelid involvement and serum cortisol, although in retrospective data only (12). Relatedly, topical steroid withdrawal (TSW) has been described as intense erythema, burning, and papulopustular eruption after cessation of prolonged TCS use, particularly on the face, but its mechanisms remain controversial and poorly defined (13–16).

Corticosteroid phobia is widespread, reinforced by these phenomena and by patient information from non-specialist sources (17). Large international surveys using the TOPICOP scale show substantial fears about TCS safety among patients and caregivers (18, 19), resulting in underdosing, self-reduction of frequency, and premature discontinuation (20). These behavioral constraints compound pharmacological ones, effectively lowering the real-world ceiling on TCS exposure.

Importantly, even when TCS are deployed according to guidelines, proactive therapy does not abolish relapses. In proactive fluticasone regimens, roughly one fifth to two fifths of adults and adolescents relapsed within 16 weeks despite twice-weekly TCS maintenance (21), and a vehicle-controlled trial in children with mild-to-moderate disease gave a residual of the same order (22). In a pediatric trial of crisaborole-based proactive maintenance, almost half of participants relapsed within 16 weeks following a TCS induction phase (23), and in a 52-week vehicle-controlled maintenance study most enriched responders flared at least once within the year (24). Where a non-steroidal agent has been used as needed rather than on a fixed schedule, more than half of patients experienced disease worsening within the year, although control was regained: in a pooled analysis of as-needed ruxolitinib cream the median time to worsening to IGA 2 or above was 36.1 weeks, IGA 0/1 was quickly recaptured, and patients spent a median 43.9% of the as-needed period off treatment (25), while 74.1% to 77.8% of patients were at IGA 0/1 at Week 52 (26). Thus, both steroidal and non-steroidal proactive strategies leave a sizeable residual relapse fraction over months (7). Because that fraction is of the same order whether maintenance is steroidal or non-steroidal, relapse rate alone cannot distinguish a steroid-primed mechanism from steroid-independent relapse: it defines the unmet need rather than supporting the hypothesis. The phenomena that have no non-steroidal counterpart, steroid-associated rosacea-like dermatitis and TSW, carry that discriminating weight instead.

In parallel, therapeutic innovation has focused on second-line non-steroidal agents-calcineurin inhibitors, phosphodiesterase-4 inhibitors, and more recently JAKi and aryl hydrocarbon receptor agonists-tested primarily in patients deemed unsuitable for or uncontrolled by TCS (27–29). Indirect comparisons rank several of these agents highly on short-term endpoints (30, 31), and one has been compared directly with a corticosteroid in a dose-ranging study (32). Many of these agents are effective and clinically useful, but they do not directly address how TCS themselves may load risks during otherwise successful therapy. If the main limitation of TCS is not lack of efficacy but the emergence of iatrogenic pathology and rebound at longer durations, then the most impactful innovation may be an add-on strategy that neutralizes steroid-specific liabilities while preserving their first-line role (33).

The clinical observations motivating this hypothesis include: (i) steroid-associated rosacea-like dermatitis and TSW after prolonged TCS use (10, 11, 14, 16); (ii) persistent subclinical inflammation and barrier abnormalities even in clinically normal-appearing AD skin (34–36); (iii) relapse despite proactive steroidal or non-steroidal maintenance (7, 21–24), which frames the unmet need; and (iv) clinical impressions of reduced responsiveness or residual relapse to TCS in some settings, which controlled studies in psoriasis have not confirmed (37, 38). They are not assumed to have one cause. Persistent type 2 inflammation, barrier dysfunction, tissue-resident immune cells, microbial factors, and treatment adherence provide alternative explanations for recurrence. Those results cannot establish an AD-specific mechanism but argue against presuming universal loss of TCS efficacy. Throughout, damage-associated molecular patterns (DAMPs) refer to endogenous molecules released or exposed by stressed or injured tissue that can initiate innate immune responses (39). The proposed model asks whether glucocorticoid-sensitive inflammasome signaling, potentially involving different sensors in different skin compartments, could converge on caspase-1 and reduce functional GR. This is a mechanistic extrapolation, not an established explanation for AD relapses.

## 2 Evidence synthesis

This work is a perspective and hypothesis-generating article. It synthesizes existing published data to generate a testable model and does not involve new human or animal experiments. Four evidence streams inform the synthesis:

1. Clinical trial data on TCS and non-steroidal topical agents in AD, with emphasis on induction regimens, proactive maintenance strategies, relapse rates, and cutaneous safety profiles.
2. Observational and regulatory reports on steroid-induced rosacea-like dermatitis, TSW, and perceptions of corticosteroid “tachyphylaxis,” including systematic reviews and pharmacovigilance data when available.
3. Mechanistic studies in myeloid cells, keratinocytes, and mouse models addressing glucocorticoid effects on NLRP3 inflammasome components, purinergic ATP–P2X7R signaling, LL-37 and TLR2 pathways, and itch–scratch-related DAMPs signaling in skin.
4. Translational studies on inflammasome activity, caspase-1-mediated GR cleavage, and glucocorticoid resistance in hematologic malignancies, nephrotic syndrome, asthma, chronic rhinosinusitis with nasal polyps, depression, and inflammatory dermatoses.

The narrative synthesis draws on electronic literature searches supported by AI-assisted retrieval, reference-list screening and authoritative guidelines. Topics included combinations of “atopic dermatitis,” “topical corticosteroid,” “inflammasome,” “NLRP3,” “P2X7,” “LL-37,” “caspase-1,” “glucocorticoid receptor,” “steroid resistance,” “rosacea,” and “topical steroid withdrawal.”

Evidence is classified by what each study demonstrates, not by whether it is compatible with the model. “Demonstrated in the original system” identifies experimental findings together with their cell type, species, and exposure context; it does not imply demonstration in TCS-exposed AD epidermis. “Indirect support” identifies related observations that do not test the proposed causal link. “Untested hypothesis” identifies the extrapolations that require direct validation. **Table 1** applies this classification, and **Figure 1** illustrates the proposed GR-cleavage mechanism, candidate NLRP3 branch, and conditional add-on strategy. The first proposed component is conditional glucocorticoid-dependent sensitization of an inflammasome pathway in an identified skin compartment. NLRP3 is a candidate because of the myeloid priming data, not because its dominance in human keratinocytes has been established. The second proposed component is activation by disease- or injury-associated signals during treatment or after dose reduction, with NLRP1 and NLRP3 considered separately. The third is caspase-1-dependent cleavage of GR within the affected GR-expressing cell, leading to impaired response at equivalent local glucocorticoid exposure. None of these linked events have been established as a complete sequence in TCS-exposed human AD skin.

**Table 1:** Evidence boundaries of the proposed model.

Link/observation	Evidence and original system	Not established in TCS-exposed AD skin
Glucocorticoid → NLRP3/ATP sensitization	GR-dependent responses in differentiated macrophage systems (40); neuroimmune support (41–43).	Direction and magnitude in skin; keratinocyte priming; persistence after tapering.
Glucocorticoid → inflammasome suppression	Pro-IL-1β suppression and timing-dependent inhibition in macrophages (44–46).	A skin exposure window in which priming outweighs suppression.
CCL20/TLR2/LL-37 observations	Keratinocyte responses, antimicrobial-peptide suppression, and separate rosacea studies (47–57).	A single steroid-induced cutaneous amplification sequence.
NLRP1 → cytokine release	Human keratinocyte and AD-associated studies, including *S. aureus* challenge and NLRP1 silencing (58, 59).	Steroid-induced NLRP1 priming, P2X7R dependence, or GR cleavage.
Dermal Myeloid contribution to AD	Human dermal macrophage/inflammasome and spatial-network observations (60, 61); topical/oral TDCA mouse studies (62, 63).	Macrophage-exclusive causality, TCS-induced priming, GR cleavage, or reversal of steroid resistance.
GPCR19–P2X7R–NLRP3 modulation	microglial and knockout experiments in Alzheimer disease models (64).	The same response in dermal myeloid cells; a glucocorticoid challenge or GR-preserving effect.
Caspase-1 → GR cleavage/resistance	Leukemia and recombinant-protein experiments; perturbation and rescue (65, 66).	Cleavage in viable skin cells at clinically relevant local exposure.
Residual relapses during maintenance	Clinical TCS and nonsteroidal maintenance studies (7, 21–26).	A shared inflammasome driver or a TCS-specific pharmacodynamic ceiling.
Integrated pathway/adjunct benefit	Linked interpretation of separate published observations.	The complete same-cell sequence, clinical frequency, GR preservation, or longer safe TCS use.

**Figure 1:**
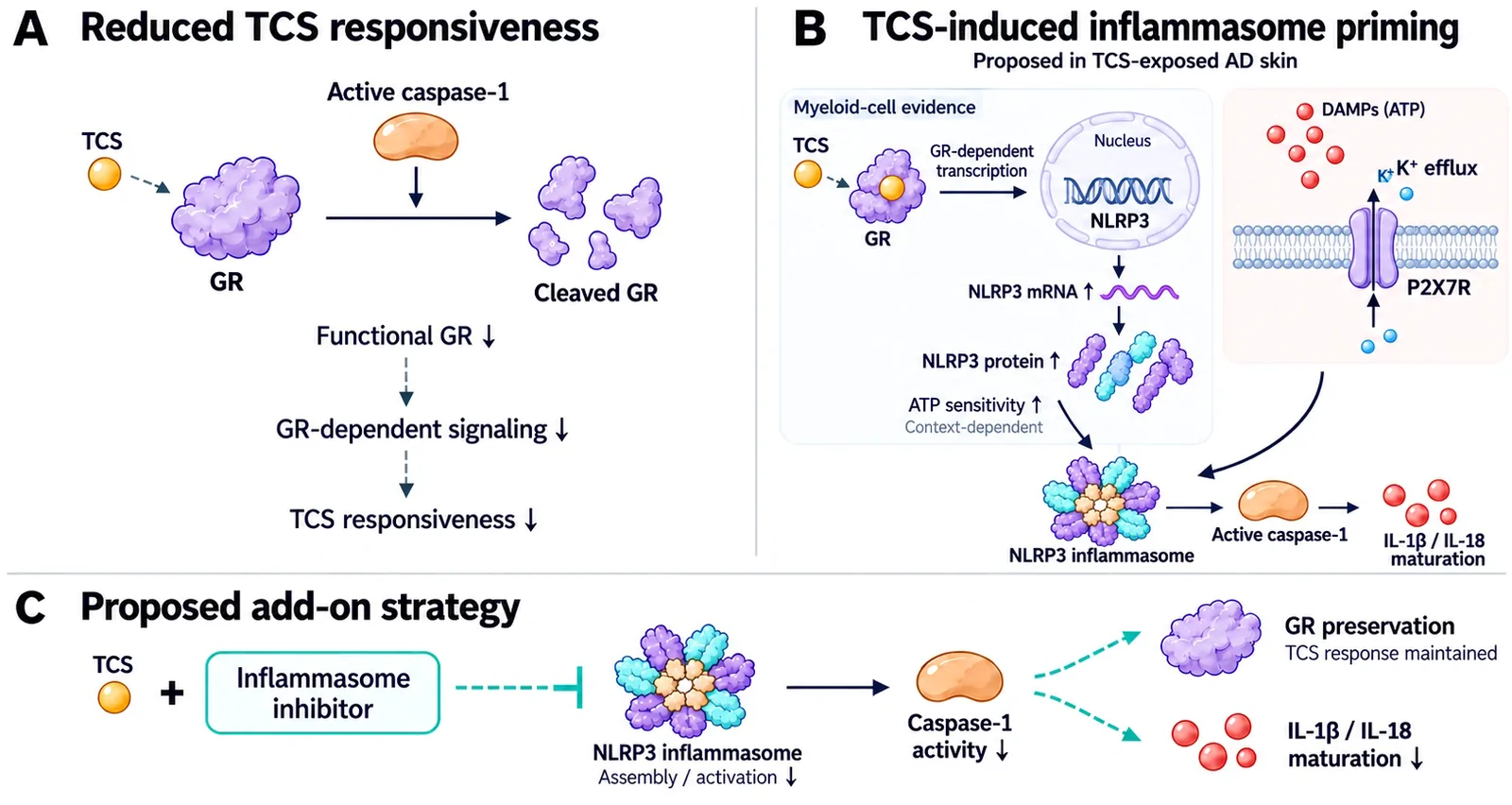
Proposed steroid-primed inflammasome–caspase-1–GR axis. Solid arrows mark steps demonstrated in the experimental system; dashed arrows mark proposed steps that have not been demonstrated in TCS-exposed human skin. **(A)** Reduced TCS responsiveness. Active caspase-1 cleaves the GR, reducing the pool of intact receptor; the consequent fall in GR-dependent signaling and in responsiveness to an equivalent glucocorticoid exposure is the proposed pharmacodynamic ceiling. **(B)** TCS-induced inflammasome priming, proposed in TCS-exposed atopic dermatitis skin. Left panel, glucocorticoids acting through GR-dependent transcription raise NLRP3 mRNA and protein and increase sensitivity to extracellular ATP in macrophage systems (40). Right panel: DAMPs, principally extracellular ATP, act on P2X7R with K+ efflux to supply the activation signal by itch-scratch cycle etc. (67–69). The two inputs converge on the NLRP3 inflammasome, activating caspase-1 and driving IL-1β and IL-18 maturation. NLRP3 is drawn as the candidate branch because the glucocorticoid-priming data exists for it; NLRP1 is a complementary upstream route in human keratinocytes, including the *S. aureus*-associated response (59), and is not shown. **(C)** Proposed add-on strategy. An inflammasome inhibitor co-administered with TCS from induction onward is proposed to reduce inflammasome assembly and caspase-1 activity, thereby preserving intact GR and maintaining the TCS response while lowering IL-1β and IL-18 maturation. The intervention targets the convergent node and is therefore independent of which sensor is upstream.

No formal systematic review or meta-analysis was attempted, and risk-of-bias tools were not applied, given the mechanistic and conceptual focus. Consequently, clinical associations and findings from separate experimental systems cannot be combined to estimate the frequency, magnitude, or clinical impact of the proposed mechanism.

## 3 The proposed model

### 3.1 Glucocorticoids as paradoxical primers of the inflammasome

Human skin expresses both NLRP1 and NLRP3, although their expression and functional contributions vary with cellular compartment and inflammatory context (58, 70). Sand et al. (58) demonstrated NLRP1-dependent IL-1β and IL-18 release following UVB irradiation of human primary keratinocytes, together with substantial differences in inflammasome expression and responsiveness between human and murine keratinocytes. Vaher et al. (59) reported increased NLRP1 protein expression and caspase-1 activity in AD skin and showed that NLRP1 silencing reduced *S. aureus*-induced IL-1β and IL-18 production in keratinocytes. Evidence also supports NLRP3 expression and signaling in AD-related settings, although the findings are context dependent: Niebuhr et al. (70) observed reduced NLRP3 and caspase-1 expression in lesional AD skin and downregulation of NLRP3 and ASC by Th2 cytokines in primary human keratinocytes, whereas Ramos et al. (60) reported increased dermal expression of both NLRP1 and NLRP3 in severe AD, accompanied by increased ASC, caspase-1, and gasdermin D expression and IL-1β-positive macrophages. Ghaderpour et al. (62) further reported P2X7R-associated NLRP3 inflammasome signaling in cultured human keratinocytes, including inflammasome component expression, NLRP3 oligomerization, and mature IL-1β and IL-18 production. NLRP3-dependent IL-1β production by basophils in a murine AD-like model provides additional evidence in a distinct cellular compartment (71). Collectively, these findings support evaluating both NLRP1 and NLRP3 while distinguishing sensor expression from functional activation; they do not establish a universal hierarchy between the two sensors or demonstrate TCS-induced epidermal inflammasome priming (59, 60, 62, 70).

Human AD lesions contain a disease-relevant dermal inflammatory compartment: Ramos et al. (60) reported increased dermal inflammasome-related signals and CD68-positive macrophages associated with ASC, gasdermin D, and IL-1β in moderate-to-severe AD. Spatial and single-cell profiling localized CCL13/CCL18-expressing macrophages and LAMP3-positive dendritic cells expressing CCL17/CCL22 within upper-dermal inflammatory networks containing T cells and fibroblasts (61). These observations support myeloid contributions to AD inflammation and immune-cell recruitment, although spatial association and inferred interactions do not alone prove causality. Myeloid populations are heterogeneous; neither uniform inflammasome activation nor universal steroid resistance is assumed.

In myeloid cells, glucocorticoids can directly upregulate NLRP3 expression and sensitize cells to ATP-induced inflammasome activation. Dexamethasone and cortisol induce NLRP3 transcripts and protein in THP-1-derived macrophages via GR-dependent transcriptional mechanisms, without altering ASC or caspase-1 levels, and increase ATP-driven IL-1β release in an NLRP3- and caspase-1-dependent manner (40). Similar priming phenomena have been observed in microglia and other neuroimmune cells, in which chronic glucocorticoid exposure increased NLRP3 mRNA and potentiated responses to subsequent proinflammatory stimuli (41, 42). This effect has been linked, at least in part, to thioredoxin-interacting protein (TXNIP) (43). These observations establish context-specific effects, not a general proinflammatory action of TCS. The context-dependent candidate priming pathway is summarized in **Figure 1**. The priming hypothesis is restricted to conditions in which sensitization can be demonstrated experimentally. No clinically applicable priming window is known for TCS-exposed human epidermis, so the model is committed here to a prediction that can be measured rather than to an open possibility: sensitization is predicted to occur only when the free intracutaneous glucocorticoid concentration is sub-saturating for the glucocorticoid receptor (GR)—that is, at or below the 100 nM dexamethasone concentration that primes myeloid cells (40) —and lies on the ascending limb of the corticosterone concentration–response curve. In RAW264.7 macrophages, this ascending limb corresponds to corticosterone concentrations that enhance LPS-induced NLRP3 expression at 300 ng/mL (~0.87 µM), whereas concentrations above 700 ng/mL (~2.0 µM) suppress NLRP3 expression (46). Because these two glucocorticoids differ in GR affinity, the numerical values are not interchangeable between systems; each bounds the window only within the system in which it was measured. For example, Busillo et al. (40) used 100 nM dexamethasone in macrophage experiments, whereas Diaz-Jimenez et al. (45) observed inhibitory effects with 100 nM dexamethasone after prolonged LPS priming. The shared nominal concentration does not establish equivalent biology: cell state, treatment sequence, challenge, and duration differ. Nominal culture concentrations are not free intracutaneous concentrations, so this prediction has to be tested against measured free steroid levels in viable epidermis and dermis, not against applied potency or vehicle concentration. Furthermore, in a separate human AD/keratinocyte study, *S. aureus*-associated IL-1β and IL-18 release was NLRP1-dependent (59).

Opposing evidence is integral to the model. At the same time, glucocorticoids can suppress pro-IL-1β transcription and IL-1β secretion (44, 72), which has led to the dominant view of glucocorticoids as overall suppressors of inflammasome activity (72). These apparently conflicting actions can be reconciled by distinguishing sensor abundance (NLRP3), signal threshold (ATP sensitivity), and cytokine substrate availability (pro-IL-1β). Under this view, glucocorticoids may raise the capacity and sensitivity of the inflammasome while transiently reducing its output under continued drug exposure (40, 44). Consistent with a threshold effect rather than a uniform one, corticosterone raised lipopolysaccharide-induced NLRP3 expression at a lower concentration and lowered it at higher concentrations in murine macrophages (46), and the direction of the glucocorticoid effect in macrophages depends on when the hormone is given relative to the priming stimulus (45). This is the principal vulnerability of the model and is stated as a falsification condition rather than reconciled away. TCS are applied to achieve high, sustained local concentrations, which is the regime in which these same experiments show suppression; the model therefore requires that a sub-saturating compartment exists in treated skin. The candidate settings are early exposure before steady state, the tapering tail, and the concentration gradient at the margins of the treated field and in the deeper dermis, where free concentration may fall below the suppressive range while exposure continues. If free intracutaneous glucocorticoid concentrations under clinically used regimens are measured and found to lie in the suppressive range throughout induction and maintenance, and inflammasome sensitization cannot be demonstrated at those concentrations in human skin, the model fails.

Indirect cutaneous support: In keratinocytes, glucocorticoids can also exert proinflammatory effects. Dexamethasone induces the chemokine CCL20 in primary human keratinocytes and in mouse skin in a GR-dependent manner, with greater induction under inflammatory conditions (47). CCL20 recruits CCR6-positive Th17 cells and dendritic cells (73). Th17 cells have been implicated in the pathogenesis of atopic dermatitis, infiltrating the papillary dermis and correlating with disease severity (74), and IL-22 acts alongside the type 2 cytokines as a pathogenic axis in the disease (75). Th17 and Th22 cytokines in turn activate NLRP3, caspase-1, and IL-1β/IL-18 release in keratinocytes via ROS-dependent mechanisms (76); reciprocally, caspase-1-processed IL-1β and IL-18 promote IL-17 production by γδ and CD4 T cells (77). Thus, glucocorticoid-induced CCL20 may promote a Th17/Th22-inflammasome amplification loop in the epidermis.

Although direct evidence for GR-dependent NLRP3 induction in keratinocytes is lacking, the combination of myeloid data (40) and keratinocyte CCL20 induction (47) supports the plausibility of glucocorticoid-sensitive inflammasome circuitry in which human keratinocytes-unlike murine ones-express and assemble functional inflammasomes (58). Nonetheless, the CCL20 observations do not demonstrate direct GR-dependent NLRP3 induction, inflammasome assembly, or GR cleavage in keratinocytes. Accordingly, macrophage and microglial findings cannot be assigned to epidermal keratinocytes without further cell-specific testing.

### 3.2 Loading a TLR2–LL-37–ATP amplifier during TCS therapy

Untested temporal hypothesis: TCS exposure might increase the response to a subsequent DAMPs signal in some compartments despite concurrent anti-inflammatory effects.

Glucocorticoids modulate TLR2 expression in keratinocytes and epithelial cells. Dexamethasone can increase TLR2 expression in primary human keratinocytes, mainly in the undifferentiated state (48), and in airway epithelial cells glucocorticoids and tumor necrosis factor alpha cooperatively regulate TLR2 transcription through a glucocorticoid-responsive promoter element (49); in normal human epidermal keratinocytes, dexamethasone enhanced *Cutibacterium acnes*-induced TLR2 expression at both the transcript and the cell-surface level (50). In rosacea, TLR2 expression is increased and stimulates enhanced production of serine proteases such as kallikrein-5 by keratinocytes (51), and serine protease activity and cathelicidin are elevated in lesional skin (52), with kallikrein-5 processing the cathelicidin precursor hCAP18 into LL-37 (53). LL-37, in turn, triggers skin inflammation through NLRP3-dependent pathways, and NLRP3 inhibition substantially attenuates LL-37-induced rosacea-like pathology in experimental models (54, 55); LL-37 also sustains its own production through a TLR2-mTORC1 feedback loop in keratinocytes (56).

In AD, short-term TCS treatment suppresses epidermal antimicrobial peptides, including LL-37, to below levels observed in healthy skin (57). Thus, during active TCS therapy, ligand supply (LL-37) and some inflammatory outputs are reduced even as receptor-side sensitivity (TLR2) and potential inflammasome capacity may be increasing (40, 48). After TCS tapering or withdrawal, barrier disruption (78), microbial stimuli — *S. aureus* colonization drives inflammasome-dependent IL-1β and IL-18 release in AD keratinocytes, reported there as NLRP1-dependent (59) —, and restored cathelicidin processing (52) can rapidly replenish LL-37, thereby discharging a circuit whose sensitivity was pharmacologically raised during the treatment phase.

Barrier studies under proactive topical anti-inflammatory regimens illustrate this tension. In a randomized, observer-blind mechanistic study of individuals with quiescent AD, twice-weekly betamethasone valerate 0.1% cream preserved epidermal barrier function and stratum corneum integrity but significantly increased skin-surface pH and reduced stratum corneum cohesion (79). By contrast, twice-weekly tacrolimus 0.1% ointment improved stratum corneum integrity, hydration, and protease activity (79). These biophysical changes may influence protease activity or microbial ecology, although those downstream consequences were not directly evaluated in that study.

Extracellular ATP provides a canonical “signal 2” for NLRP3 activation via P2X7R-mediated potassium efflux (67, 68, 80, 81). P2X7R is expressed and functional in the epidermis, as reviewed for skin biology and disease (82): murine epidermal Langerhans cells and keratinocytes express functional receptors (83), and in human keratinocytes purinergic stimulation modulates chemokine expression, with intense P2X7 immunoreactivity confined to the basal layer in lesional psoriasis and AD (69), and P2X7R is required for full contact hypersensitivity responses (84); the receptor also interacts physically with the NLRP3 scaffold (85) and drives NLRP3-dependent IL-1β secretion in myeloid cells (86). Mechanical injury, barrier disruption, and ROS-mediated pannexin channel opening promote ATP release from keratinocytes and other skin cells (78, 87). Gasdermin D pore formation can mediate rapid, transient ATP release after NLRP3 inflammasome activation and before lytic cell death (88). The released ATP could, in principle, provide a feed-forward signal for P2X7R-dependent inflammasome activation; however, this possibility has not been demonstrated in corticosteroid-exposed AD skin (67, 68, 80, 81, 88).

Together, these findings support the possibility that, during TCS therapy, receptor-side responsiveness and inflammasome sensor abundance may change while ligand supply and cytokine outputs are transiently constrained (40, 44, 48, 57). We hypothesize that, after tapering or withdrawal, barrier disruption, scratching, microbial stimuli, and recovery of cathelicidin processing could restore signals capable of activating a previously sensitized inflammasome circuit.

In a permissive cell state, glucocorticoid-dependent NLRP3 induction could lower the threshold for ATP-triggered inflammasome activation despite suppression of other inflammatory programs; differentiated macrophage experiments provide the component precedent (40).

### 3.3 A steroid-primed inflammasome under itch–scratch conditions

Keratinocytes and skin-resident immune cells form a functional NLRP3-ASC-caspase-1 module (58, 89). Experimental models of allergic contact dermatitis show that contact allergens increase NLRP3 and ASC protein expression in human keratinocytes, promote recruitment of ASC to the inflammasome, and activate caspase-1 (90), and that contact sensitizers drive caspase-1/ASC-dependent IL-1β and IL-18 processing and secretion in human keratinocytes, with impaired responses in ASC- and NLRP3-deficient mice (89). Comparable inflammasome activity is observed in human AD skin, where ASC and gasdermin D activation and IL-1β-secreting macrophages have been documented in severe disease (60), and in murine AD-like inflammation initiated by IL-33-primed basophils (71).

Clinically normal-appearing AD skin is not immunologically normal. Nonlesional skin displays barrier defects and immune abnormalities (35) and clonal T cells shared with lesional skin (36), and these abnormalities can persist after clinical improvement; subclinical inflammation is detectable before relapse (34). Long-lived IgE plasma cells (91) and FcϵRI-bound IgE with a prolonged tissue half-life (92) provide stable humoral drivers of ongoing TH2 activity. Thus, even under apparent remission, a low-grade inflammatory milieu persists, detectable as altered cytokine concentrations across the stratum corneum (93).

TH2 cytokines can suppress NLRP3 and ASC expression in keratinocytes, which may restrain inflammasome activity in untreated AD (70). However, if glucocorticoids directly or indirectly prime NLRP3 and associated pathways (40, 47), they may partially override this TH2-mediated brake, especially under proactive dosing to clinically quiescent but immunologically active skin (34).

The itch–scratch cycle is a plausible source of injury-associated signals, but published hapten-induced ATP-release experiments do not measure ATP during scratching or TCS tapering in patients (87). Itch driven by TH2-associated mediators leads to scratching, which further disrupts the barrier and releases ATP, ROS, and other DAMPs (78, 87). Haptens and irritants can open pannexin hemichannels and promote ATP release from keratinocytes (87), and ATP then activates P2X7R, a potent NLRP3 trigger (67, 68). *S. aureus* colonization, prevalent in AD, contributes additional inflammasome stimuli via NLRP1 and other sensors and promotes IL-1β and IL-18 release (59).

In the context of prior TCS exposure, these chronic, variably intense DAMPs signals could episodically activate a sensitized inflammasome circuit and contribute to inflammatory flares. Whether TCS exposure produces this sensitized state in human AD skin remains to be established (40, 47, 48).

### 3.4 Caspase-1–mediated GR cleavage

Studies in acute lymphoblastic leukemia demonstrate that caspase-1 can directly cleave GR and confer glucocorticoid resistance (65). In resistant blasts, CASP1 and NLRP3 promoters are hypomethylated and overexpressed. Caspase-1 overexpression leads to GR cleavage, reduced occupancy at glucocorticoid response elements, and reduced transcriptional responses to glucocorticoids, whereas caspase-1 inhibition or expression of cleavage-resistant GR mutants restores steroid sensitivity (65). This establishes a mechanistic link between inflammasome activity and the number of functional GR molecules available, effectively creating a “receptor rheostat” (66).

If the resulting active caspase-1 cleaves GR within the same viable dermal macrophage or monocyte-derived cell, subsequent GR-dependent transcription and anti-inflammatory responses could decline at equivalent intracellular steroid exposure. This receptor-level step is extrapolated from leukemia studies, not demonstrated in normal dermal myeloid cells (65, 66). The hypothesis therefore prioritizes the sequence TCS exposure → context-dependent myeloid priming → inflammasome activation → same-cell GR cleavage → reduced steroid response, with each arrow requiring validation (**Figures 1A, B**). Crucially, caspase-1 activity in a dermal myeloid cell would not itself demonstrate direct cleavage of GR in a neighboring keratinocyte. Caspase-1 activity, GR cleavage, and impaired GR function need to be localized to the same cell population, or a separate intercellular mechanism needs to be demonstrated.

Inflammasome-related signatures also associate with steroid resistance in other conditions, including idiopathic nephrotic syndrome (94), certain asthma endotypes (95), chronic rhinosinusitis with nasal polyps (96), and treatment-resistant depression (97). These observations support the concept that NLRP3-caspase-1 activity can contribute to tissue-specific glucocorticoid resistance across diseases, although outside the leukemia work they remain associations rather than demonstrations of cleavage.

Untested cutaneous consequence: If reproduced in corticosteroid-exposed skin, this mechanism could link inflammasome activation to both inflammatory outputs—including IL-1β and IL-18 maturation and, in relevant cellular contexts, pyroptotic signaling—and progressive loss of intact GR through caspase-1-mediated cleavage (60, 65, 88, 98). During ongoing TCS therapy, GR-mediated transrepression may still control downstream cytokine production (44, 72), preserving clinical remission. Over time, however, cumulative inflammasome activity could deplete functional GR to the point that a constant or even lower intensity of underlying disease drivers produces overt relapse, flare, or iatrogenic dermatitis at the same TCS dose. Nonetheless, direct measurement of cleavage products and GR function is required to distinguish this possibility from inflammation-associated changes in cell composition or nonspecific cell death.

Competing mechanisms must be considered in parallel. Classical mechanisms of reduced steroid responsiveness remain relevant in the skin: GRβ is increased in peripheral-blood lymphocytes of patients with severe AD unresponsive to topical corticosteroids (99) and both isoforms are expressed in inflammatory dermatoses (100), while HDAC2-dependent repression has been implicated in ultraviolet-B-induced glucocorticoid resistance in keratinocytes (101). The caspase-1 mechanism adds an upstream, inflammasome-driven determinant that links TCS exposure directly to GR loss and limiting prolonged TCS usage (65, 66). However, a decrease in total GR alone might not be evidence of caspase-1 cleavage. Concordant detection of specific fragments, reduced GR-dependent function, and rescue by orthogonal CASP1 perturbation would be needed to attribute a receptor-level effect to this mechanism.

At the tissue level, persistent myeloid cytokine and chemokine output could sustain neighboring immune-cell activation and epidermal inflammation even when keratinocyte GR remains intact. This is a proposed paracrine consequence of compartment-restricted resistance, not a claim that myeloid caspase-1 directly cleaves GR in adjacent epidermal cells. Human dermal network studies and the published taurodeoxycholate studies provide disease-relevance and intervention precedents for this interpretation (60–62), but persistent cytokine production alone cannot distinguish receptor-level resistance from strong ongoing stimulation, inadequate dermal drug delivery, or a normally steroid-insensitive output. Neither all dermal myeloid populations nor all AD lesions are expected to share the same response state.

### 3.5 Therapeutic perspective: conditional intervention points

The model identifies experimental intervention points rather than established treatment recommendations. The integrated model delineates three potential points of intervention:

#### 3.5.1 Priming (signal 1)

Modulating GR-dependent transcriptional priming of NLRP3 and related components could limit future inflammasome assembly (40, 43). However, indiscriminate suppression of GR activity risks attenuating beneficial anti-inflammatory effects (72) and compromising epidermal barrier function (102, 103).

#### 3.5.2 Inflammasome assembly and activation

Targeting NLRP3, P2X7R, or upstream ATP signaling may reduce caspase-1 activation and IL-1β/IL-18 release (55, 67, 68, 86). NLRP3 inhibition attenuates LL-37-driven rosacea-like inflammation in experimental systems (54), and a GPCR19 agonist that restrains inflammasome signaling ameliorated AD-like disease in mice (62), supporting the plausibility of this approach. But a negative result would not exclude an NLRP1-dependent route (59).

#### 3.5.3 Caspase-1 effector activity

Direct inhibition of caspase-1 is uniquely positioned to affect both inflammatory output and GR preservation (65, 66). If caspase-1-mediated GR cleavage is reproduced in corticosteroid-exposed skin, caspase-1 inhibition would be predicted to preserve GR integrity and maintain corticosteroid responsiveness across repeated TCS cycles. The conditional add-on strategy is summarized in **Figure 1C**.

Because epidermal GR is critical for barrier integrity and cutaneous homeostasis (102, 103), preserving GR function may yield dual benefits: sustained anti-inflammatory responsiveness and improved barrier competence. Notably, in cell models, simple GR overexpression does not overcome caspase-1-driven resistance unless caspase-1 is inhibited (65), implying that escalation of TCS potency or dose alone may be insufficient to surmount a caspase-1-dominated ceiling.

## 4 Discussion

This article proposes a possible, context-dependent inflammasome–caspase-1–GR mechanism, not an established ceiling on TCS treatment duration. Specifically, it proposes that the limit on TCS duration in AD is, in part, a pharmacodynamic quantity determined by the interaction between TCS and a steroid-primed P2X7R–NLRP3–caspase-1–GR axis in the skin. In this framework, TCS simultaneously suppress and prepares a system that can later discharge as inflammatory rebound and progressive loss of steroid responsiveness. However, the evidence supports individual components in distinct systems, whereas their temporal and cell-specific connection in human AD skin remains the central unresolved question.

The model offers one possible explanation for how TCS can remain acutely effective while being associated, in some clinical settings, with steroid-induced rosacea-like dermatitis and topical steroid withdrawal (TSW) (10, 14). During treatment, GR-mediated transrepression may suppress cytokine outputs, whereas glucocorticoids can increase NLRP3 expression in myeloid cells and alter keratinocyte pathways including TLR2 and CCL20 (40, 47, 48). I hypothesize that, after tapering or withdrawal, DAMP signals associated with barrier disruption, scratching, microbial stimuli, LL-37, and extracellular ATP could activate a previously sensitized inflammasome circuit. This proposal has not been demonstrated in corticosteroid-exposed human AD skin. If caspase-1-mediated GR cleavage occurs in this setting, it could provide a molecular route through which cumulative inflammasome activity reduces functional GR reserve and contributes to a pharmacodynamic ceiling on subsequent TCS responses (65, 66).

The model is explicitly designed to account for iatrogenic pathology induced by TCS, rather than for steroid-naive AD pathophysiology. Studies demonstrating TH2-mediated suppression of NLRP3 and ASC in keratinocytes (70) and increased scratching in NLRP3-deficient mice (104) emphasize important nuances of AD biology in the absence of steroids. These findings are not negated by the current hypothesis; instead, they highlight that TCS add a distinct layer of regulation that may invert or override some intrinsic patterns of inflammasome activity in the skin (40, 47).

Several limitations warrant emphasis. Direct evidence for GR-dependent NLRP3 induction in human keratinocytes and for caspase-1-mediated GR cleavage in TCS-exposed skin is currently lacking; the cleavage data come from leukemia cells (65) and the induction data from myeloid cells (40). Betamethasone dipropionate has conversely been reported to inhibit NLRP3 inflammasome activation by suppressing pro-IL-1β expression (44), and glucocorticoid timing relative to priming can determine the direction of the effect in macrophages (45). Extracellular ATP and LL-37 dynamics have not been characterized across TCS induction, maintenance, tapering, and withdrawal in humans. Clinical descriptions of TSW are heterogeneous and often retrospective, and standard definitions are still evolving (14, 16). Controlled studies in psoriasis have failed to demonstrate therapeutic tachyphylaxis to topical steroids (37, 38). Classical mechanisms of steroid resistance (99–101) and other inflammasome-independent pathways may contribute substantially to individual patient trajectories. Finally, broad inhibition of inflammasome components or caspase-1 could carry trade-offs for host defense, since the same sensors are engaged by *S. aureus* in AD skin (59).

Validation of this hypothesis would require determining whether clinically relevant TCS exposures suppress or sensitize inflammasome responses in human skin models, accounting for treatment timing, cell viability, and compartment-specific exposure. Functional activation should be distinguished from transcriptional changes alone.

A cleavage-dependent mechanism would require evidence linking GR fragmentation to impaired transcription, supported by complementary CASP1 perturbations and, where feasible, cleavage-resistant GR rescue. Epidermal and dermal responses should be evaluated separately, without presuming the upstream sensor.

Longitudinal studies with appropriate controls, documented treatment exposure, and prespecified clinical criteria could clarify relevance to treatment-associated flares.

A randomized adjunctive trial would be appropriate only after sufficient mechanistic and safety evidence emerges. Clinical improvement without GR preservation would not establish cleavage dependence. These proposed investigations have not been undertaken here, and the hypothesis does not warrant departures from established TCS practice (1, 2, 8).

Nonetheless, the model yields concrete, testable predictions. Longitudinal studies of patients receiving standardized TCS regimens for AD could assess expression of NLRP3, P2X7R, caspase-1, gasdermin D, and GR (including cleavage products) in lesional and nonlesional skin during induction, proactive maintenance, tapering, and withdrawal (60, 65). Free glucocorticoid concentrations should be measured in the same specimens, by depth where feasible, because the model stands or falls on whether the exposure achieved in treated skin lies in the sensitizing or the suppressive part of the concentration-response. Measurements of extracellular ATP, LL-37, IL-1β, and IL-18 in the stratum corneum (93), coupled with high-resolution imaging of barrier function and *S. aureus* colonization (59, 79), could further clarify the dynamics of signal 2 provision. Patients developing steroid-associated rosacea-like dermatitis (10, 12) or TSW (14, 16) could be compared with matched TCS-treated controls without such phenomena to identify differential inflammasome and GR signatures.

Interventional studies could test add-on agents that target components of this axis. Topical P2X7R or inflammasome inhibitors (55, 68), or caspase-1-focused therapies (65), co-administered with standard TCS from induction onwards, could be evaluated for their ability to preserve GR integrity, reduce markers of inflammasome activation, extend relapse-free survival, and enable safer TCS tapering at equal or lower cumulative doses; a GPCR19 agonist acting on this axis has shown activity in murine AD (62). Barrier-restoring and acidifying regimens may partially mitigate ATP and LL-37-driven signaling (33, 78, 79), offering a pragmatic initial test of the model even before dedicated inflammasome inhibitors reach the clinic.

If validated, this framework would justify a shift in therapeutic strategy. Rather than primarily seeking non-steroidal substitutes to replace TCS once problems arise (28–31), the field could prioritize rational add-on approaches that protect GR and neutralize steroid-induced inflammasome priming while leveraging the high anti-inflammatory potency and accessibility of TCS (1, 8). Such an approach could prolong safer TCS usage, reduce dependence on more complex or costly therapies (105), and address patient and caregiver concerns about steroid safety in a mechanistically grounded way (18, 20).

## 5 Conclusion

This article presents a hypothesis grounded in published data, not experimental confirmation of a TCS-induced cutaneous inflammasome–GR pathway. The apparent paradox of corticosteroid-associated cutaneous inflammation may be more tractable if TCS are considered agents that both treat AD and, under exposure conditions, could alter inflammasome-related signaling (40, 47, 48). The model advanced here hypothesizes that steroid-associated rosacea-like dermatitis and TSW could involve discharge of a pharmacologically sensitized inflammasome circuit under the combined influence of barrier disruption, itch–scratch dynamics, microbial stimuli, LL-37, and extracellular ATP (52, 59, 78, 87, 88). If caspase-1-mediated GR cleavage occurs in corticosteroid-exposed skin, it could progressively reduce steroid responsiveness and limit long-term usage of TCS (65, 66).

In this framework, the limitation of prolonged TCS therapy is not purely empirical but mechanistic: cumulative inflammasome activity and GR loss are proposed to determine how long and how safely TCS can be used before the steroid-specific iatrogenic phenotypes, rosacea-like dermatitis and TSW, and reduced responsiveness emerge. Relapses that occurs at comparable rates under non-steroidal maintenance is not attributed to this mechanism. Recognizing the mechanisms underlying these limitations suggests new therapeutic opportunities: add-on strategies that specifically target priming, inflammasome assembly, or caspase-1 could preserve GR integrity and prolong TCS usage.

Given the ubiquity of TCS in AD management (1–3) and the lack of targeted tools to manage their long-term liabilities, systematic evaluation of inflammasome activity, extracellular ATP, LL-37, and GR status across the full TCS treatment cycle is a timely priority for translational research (60, 93).

## Data Availability

Publicly available datasets were analyzed in this study. This data is available in the listed references.
